# Molecular Image Segmentation Based on Improved Fuzzy Clustering

**DOI:** 10.1155/2007/25182

**Published:** 2007-10-10

**Authors:** Jinhua Yu, Yuanyuan Wang

**Affiliations:** Department of Electronic Engineering, Fudan University, Shanghai 200433, China

## Abstract

Segmentation of molecular images is a difficult task due to the low signal-to-noise ratio of images. A novel two-dimensional fuzzy C-means (2DFCM) algorithm is proposed for the molecular image segmentation. The 2DFCM algorithm is composed of three stages. The first stage is the noise suppression by utilizing a method combining a Gaussian noise filter and anisotropic diffusion techniques. The second stage is the texture energy characterization using a Gabor wavelet method. The third stage is introducing spatial constraints provided by the denoising data and the textural information into the two-dimensional fuzzy clustering. The incorporation of intensity and textural information allows the 2DFCM algorithm to produce satisfactory segmentation results for images corrupted by noise (outliers) and intensity variations. The 2DFCM can achieve 0.96 ± 0.03 segmentation accuracy for synthetic images under different imaging conditions. Experimental results on a real molecular image also show the effectiveness of the proposed algorithm.

## 1. INTRODUCTION

Molecular imaging techniques such as positron emission imaging, fluorescent imaging, and isotope radiation imaging have undergone explosive growth over the past few decades. It will allow clinicians not only to measure concentrations of interesting molecules quantitatively, but also to visualize the interactions of molecular markers in vivo, thus extending the emphasis of radiological imaging beyond the anatomical and functional levels [1]. Integrations of molecular information specific to each patient with anatomical information obtained by conventional imaging methods such as the magnetic resonance imaging (MRI), X-ray computed tomography (CT), and ultrasound (US) will undoubtedly enhance the ability to fight disease. Image segmentation is a preliminary and crucial step for subsequent image applications such as quantification of molecular concentration, image registration, and integration. However, molecular images often suffer from a low signal-to-noise ratio (SNR); this will lead to difficulties with its segmentation.

The fuzzy clustering algorithm, more widely used as fuzzy C-means algorithm (FCM) [2], has been successfully utilized in medical image segmentation [3–6]. The most important feature of the FCM is that it allows each pixel to belong to multiple clusters according to its degree of membership in each cluster, which makes the clustering methods able to retain more information from the original image as compared to the case of hard segmentation. FCM works well on images with low levels of noise, but there are two disadvantages of the FCM used in segmentation of noise-corrupted images. One is that the FCM does not incorporate the information about the spatial context, which makes it sensitive to the noise and other imaging artifacts. The other is that the cluster assignment is based solely on the distribution of the pixel intensity, which makes it sensitive to intensity variations due to the illumination or the object geometry [7]. In order to improve the robustness of conventional FCM, many algorithms have been presented in the literatures. These methods can be divided into two main groups: imposing spatial constraints to clustering algorithms [3, 5–7] and introducing other features or dissimilarity index that is insensitive to intensity variations in the objective function of FCM [5, 7].

This paper presents a novel algorithm based on fuzzy logic for molecular image segmentation. In this algorithm, two factors to improve the robustness of conventional FCM are considered. Due to the low SNR of molecular images, image denoising is taken for a prelude to the segmentation. A denoising method which combines a Gaussian noise filter with an anisotropic diffusion (AD) technique is presented to alleviate noise in molecular images. Since the Gabor wavelet representation of molecular images is relatively robust to intensity variations, a texture characterization method derived from Gabor filters bank is presented to extract texture information from images. Spatial constraints provided by the denoising data and texture information provided by the Gabor wavelet are embedded in the objective function of a novel two-dimensional fuzzy clustering (2DFCM) algorithm.

The remainder of this paper is organized as follows. Section 2 proposes the denoising method. Section 3 introduces the multichannel Gabor filters and the texture feature characterization. In Section 4, we present in detail the new two-dimensional FCM algorithm (2DFCM) which integrates both intensity information and texture information. The experimental comparisons are presented in Section 5. Section 6 concludes the paper.

## 2. MOLECULAR IMAGE DENOISING

Gaussian noise is the most common noise broadly existed in signal processing sciences. Ling and Bovik [8] proposed a method to smooth molecular images by assuming that the noise follows an additive Gaussian model. Following Ling and Bovik's notion, we also assume that molecular images are corrupted by a zero-mean Gaussian white noise.

The FIR filter is well known for its ability to remove Gaussian noise from signals but it does not work very well in the image processing since it blurs edges within the image. The Gaussian noise filter (GNF) [9], combining a nonlinear algorithm and a technique for automatic parameter tuning, is a valid method for estimation and filtering of Gaussian noise. The GNF used in this paper can be summarized as follows. Let *X* = {*x*_1_, *x*_2_, … , *x*_*n*_} be a set of *n* data points in the noisy image. The output *Y* = {*y*_1_,*y*_2_, … ,*y*_*n*_ is defined as

(1) $$y_{i}=x_{i}+\frac{1}{N_{R}}\underset{x_{r}\in N_{i}}{\sum }\varsigma (x_{r},x_{i}),\;\begin{array}{c} i=1,\ldots ,n, \end{array}$$

(2) $$\zeta (x_{i},x_{j})=\left\{ \begin{array}{ll} x_{i}-x_{j}, & \left|x_{i}-x_{j}\right|\leq p,\\ \left(\frac{3p-\left|x_{i}-x_{j}\right|}{2}\right)\text{sgn}(x_{i}-x_{j}), & p<\left|x_{i}-x_{j}\right|\leq 3p\\ 0, & \left|x_{i}-x_{j}\right|>3p, \end{array}\right.,$$

where *N*_*i*_ stands for the neighborhood configuration with respect to a center pixel *x*_*i*_, and *N*_*R*_ is the cardinality of *N*_*i*_. The automatic tuning of the parameter *p* is a key step in GNF. Let MSE(*k*) denote the mean square error between the noisy image filtered with *p* = *K* and the same image filtered with *p* = *k* − 1. A heuristic estimate of the optimal parameter value is

(3) $$\hat{p}=2(k_{m}-2),$$

where

(4) $$\text{MSE}(k_{m})=\text{MAX}\{\text{MSE}(k)\}.$$

The GNF can remove intensity spikes due to the Gaussian noise. However, it has limited effect on suppressing little intensity variations caused by the neighboring smoothing. Since the conventional FCM is a method based on the statistical feature of the image intensity, a piecewise-smooth intensity distribution will be greatly beneficial to it. We pursue a more desirable denoising result by following the GNF with an anisotropic diffusion filter. Yu and Acton [10] provided an improved anisotropic diffusion filter called speckle reducing anisotropic diffusion (SRAD) which outperforms the traditional Perona-Malik nonlinear diffusion [11]. Although SRAD is proposed for the speckle reduction in synthetic aperture radar (SAR) or ultrasound images, its advantages in mean preservation, variance reduction, and edge localization are also preferable for molecular images. The SRAD used in this paper can be formulated as a diffusive process:

(5) $$c(q)=\frac{1}{1+\left[q^{2}(x,y;t)-q_{0}^{2}(t)\right]/\left[q_{0}^{2}(t)(1+q_{0}^{2}(t))\right]},$$

where *c*(*q*) represents the diffusion coefficient, *q*(*x*, *y*; *t*) is the instantaneous coefficient of variation served as the edge detector in the noise image. *q*(*x*, *y*; *t*) combines a normalized gradient magnitude operator and a normalized Laplacian operator:

(6) $$q(x,y;t)=\sqrt{\frac{(1/2){\left(|\nabla Y|/Y\right)}^{2}-(1/4^{2}){\left(\nabla^{2}Y/Y\right)}^{2}}{{\left[1+(1/4)(\nabla^{2}Y/Y)\right]}^{2}}},$$

where *∇* is the gradient operator and *Y* is the image filtered by GNF. *q*_0_(*t*) is the scale function serving as the diffusion threshold which can be approximated by using a heuristic constant *q*_0_ with the exponential decay function

(7) $$q_{0}(t)\approx q_{0}\exp[-\rho t].$$

Here *ρ* is a constant typically set to 1/6. Suppose that the output of the SARD with *Y* = {*y*_1_, *y*_2_, … , *y*_*n*_} as the input can be represented by X* = {*x*_1_*, *x*_2_*,…, *x_n_* *}.

**Figure 1:**
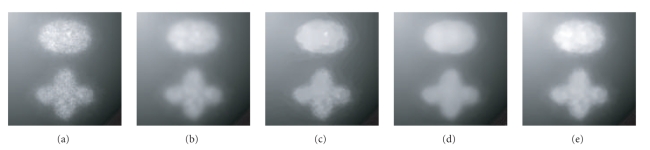
Denoising effect comparison among GNF, SRAD, and GNF plus SRAD: (a) original image; (b) filtering result of GNF; (c) filtering result of SRAD; (d) filtering result of GNF plus SRAD; (e) filtering result of anisotropic median-diffusion (MAD) [8].

To clearly illustrate the denoising effect of GNF plus SRAD, Figure 1 shows a group of filtering results of GNF alone, SRAD alone, GNF plus SRAD, and the anisotropic median-diffusion (AMD) [8] on a synthetic molecular image. From the filtering results comparison, it is seen that the denoising method of integrating GNF with SRAD can overcome the intensity fluctuation effect of GNF and the “blocky” effect of SRAD and MAD.

## 3. TEXTURE CHARACTERIZATION

A molecular image illustrates the distribution of a certain molecule [8]. Since the photon has different transportation characteristics in different turbid tissues, a molecular image can be divided into several separate regions with each region showing similar intensity (implying similar molecular concentration) and certain kind of textural pattern. Because the photon distribution in a turbid tissue is not usually uniform, the intensity within a region usually changes gradually. This intensity variation can cause errors when attempting to segment images using intensity-based classification methods. Intuitively, if a feature insensitive to the slowly varying intensity can be introduced into the classification, the performance of the image segmentation could be improved. Here, a texture characterization method based on the Gabor wavelet is utilized to obtain this desirable feature.

A large number of texture classification techniques have been proposed in the past two decades [12]. Gabor wavelet has been a popular method because it can capture the local structure corresponding to spatial frequency, spatial localization, and orientation selectivity. As a result, Gabor wavelet representation of an image should be robust to intensity variations [13, 14]. A Gabor function in the spatial domain is a sinusoidal modulated Gaussian. The real impulse response of Gabor filter is given by

(8) $$h(x,y;\mu ,\theta )=\exp\left\{-\frac{1}{2}\left[\frac{x^{2}}{\sigma_{x}^{2}}+\frac{x^{2}}{\sigma_{y}^{2}}\right]\right\}\cdot \cos(2\pi \mu x),$$

where *x* = *x* ^′^cos*θ* + *y* ^′^sin*θ*, *y* = −*x* ^′^sin*θ* + *y* ^′^cos*θ*, (*x*, *y*) represent rotated spatial-domain rectilinear coordinates, *u* is the frequency of the sinusoidal wave along the direction *θ* from the *x*-axis, *σ_x_* and *σ_y_* define the size of the Gaussian envelope along *x*- and *y*-axes, respectively, which determine the bandwidth of the Gabor filter. The frequency response of the filter is given by

(9) $$\begin{array}{ll} H(U,V) & =2\pi \sigma_{x}\sigma_{y}(\exp\left\{-\frac{1}{2}\left[\frac{{\left(U-u\right)}^{2}}{\sigma_{u}^{2}}+\frac{V^{2}}{\sigma_{v}^{2}}\right]\right\}\\ & \;\;\;\;\;\;\;+\exp\{-\frac{1}{2}\left[\frac{{\left(U+u\right)}^{2}}{\sigma_{u}^{2}}+\frac{V^{2}}{\sigma_{v}^{2}}\right]\}), \end{array}$$

where *σ*_*u*_ = 1/2π*σ*_*x*_, *σ*_*v*_ = 1/2π*σ*_*y*_. By tuning *u* and *θ*, multiple filters that cover the spatial frequency domain can be obtained. In our study, Gabor wavelets with four different scales, *μ* *∈* { *π*/ 4 $\sqrt{2}$ , *π*/ 4, *π*/ 2 $\sqrt{2}$ , *π*/ 2}, and eight orientations, *θ* ∈ {0*π*/8, 1*π*/8, … ,7*π*/8}, are used. Let *X*(*x*, *y*) be the intensity level of an image. The Gabor wavelet representation is the convolution of *X*(*x*, *y*) with a family of Gabor kernels:

(10) $$G_{\mu ,\theta }(x,y)=X(x,y)*h(x,y;\mu ,\theta ),$$

where * denotes the convolution operator, and *G*_*u,θ*_ is the convolution result corresponding to the Gabor kernel at the scale *μ* and the orientation *θ*. The next step is to compute the textural energy in *G*_*u,θ*_. The textural energy is a measure widely used to characterize the image texture. The energy that corresponds to a square window of the image *G*_*u,θ*_ centered at *x* and *y* is defined as

(11) $$E_{\mu ,\theta }(x,y)=\frac{1}{M^{2}}\sum_{(i,j)\in W_{xy}}\left|F(G_{\mu ,\theta }(i,j))\right|,$$

where *M* ^2^ is the total number of pixels in the window, and *F*(.) is a nonlinear, sigmoid function of the form

(12) $$F(t)=\tanh(\alpha t)=\frac{1-e^{-2\alpha t}}{1+e^{-2\alpha t}},$$

where *α* equals 0.25. The texture feature image is finally given by

(13) $$T(x,y)=\frac{1}{32}\sum_{\mu ,\theta }E_{\mu ,\theta }(x,y).$$

**Figure 2:**
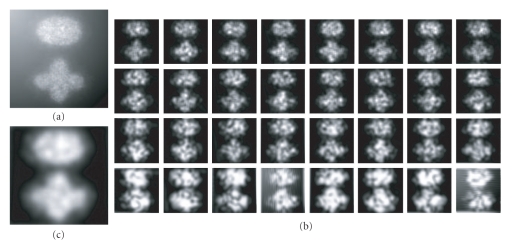
Illustration of the texture feature characterization: (a) original image with the intensity inhomogeneity; (b) texture energy bank illustration; (c) texture feature image.

As an example, Figure 2(a) shows a synthetic image with the intensity inhomogeneity. Figure 2(b) gives the texture energy bank (*E*_*μ,θ*_) illustration. Figure 2(c) shows the texture feature image. From this example, it is seen that the texture feature characterization using Gabor wavelet is insensitive to the intensity inhomogeneity.

## 4. 2DFCM

### 4.1. FCM

Let *X* = {*x*_1_, *x*_2_, …, *x*_*n*_} be a set of *n* data points, and let *c* be the total number of clusters. The objective function of the FCM [2] for partitioning *X* into *c* clusters is given by

(14) $$J_{\text{FCM}}=\overset{c}{\underset{j=1}{\sum }}\;\overset{n}{\underset{i=1}{\sum }}\mu_{ij}^{b}{\left\|x_{i}-m_{j}\right\|}^{2},$$

where *m*_*j*_, *j* = 1,2, …, *c*, represent the cluster prototypes and *μ*_*ij*_ gives the membership of pixel *x_i_* in the *j*th cluster *m_j_*. The parameter *b* is the fuzzy index that satisfies *b* ∈ (1, ∞) and controls the degree of “fuzziness” in the resulting classification. The fuzzy partition matrix satisfies

(15) $$U=\left\{\mu_{ij}\in \left[0,1\right]|\sum_{j=1}^{c}\mu_{ij}=1\;\;\forall i,\;\;0<\sum_{i=1}^{N}\mu_{ij}<N\;\forall j\right\}.$$

Under the constraints condition of (15), taking the first derivations of (14) with respect to *μ_ij_* and *m_j_* and setting those equations to zero yield necessary conditions for (14) to be minimized. Performing iteration through these two necessary conditions leads to an iterative scheme for minimizing the objective function. The objective function (14) is minimized when high membership values are assigned to pixels whose intensities are close to the centroid of its particular class, and low membership values are assigned when the pixel data is far from the centroid [2]. After FCM clustering, a segmentation of the image can be obtained by assigning each pixel solely to the class that has the highest membership value for that pixel.

Although the membership allows a pixel to deviate from multiple cluster prototypes, the spatial correlation between adjacent pixels is not considered.

### 4.2. FCM with spatial constraints

A popular method to introduce the local spatial context into the pixel classification is the spatial constraint. The spatial constraint is to let the spatial information influence the classification of the pixel of interest [5, 6]. Let *N_i_* denote the configuration of neighbors that exists in a window around *x_i_*. According to the assumption that real-world images usually have strong correlation among neighboring pixels, if the pixel *x_i_* belongs to the cluster with the prototype *m_j_*, then pixels in *N_i_* and the center pixel *x_i_* should have similar and high membership values in *m_j_*. This original idea of incorporating local spatial constraints in the FCM is formulized as [15]

(16) $$\begin{array}{ll} J_{\text{FCM}\_S} & =\overset{c}{\underset{j=1}{\sum }}\overset{n}{\underset{i=1}{\;\sum }}\mu_{ij}^{b}{\left\|x_{i}-m_{j}\right\|}^{2}\\ & \;+\frac{\alpha }{N_{R}}\overset{c}{\underset{j=1}{\sum }}\overset{n}{\underset{i=1}{\;\sum }}\mu_{ij}^{b}\left(\underset{x_{r}\in N_{i}}{\sum }{\left\|x_{r}-m_{j}\right\|}^{2}\right), \end{array}$$

where *N_i_* stands for the neighborhood configuration with respect to a center pixel *x_i_*, *N_R_* is the cardinality of *N_i_*, *α* controls the effect of the neighboring penalty. The second term on the right side of (16) allows the labeling of a pixel to be influenced by the labels in its immediate eight neighborhoods and aims at keeping continuity in the neighboring window. The problem with (16) is that computing the neighborhood terms will cost much more time than clustering. In order to reduce the complexity of computing the neighborhood terms, the dissimilarity measurements between the whole neighborhood configuration and the prototype *m_j_* can be replaced by a distance from a feature data of *N_i_* to *m_j_*. The feature data of the neighborhood configuration can be obtained by several kinds of neighboring window filters, such as the linear filter or the median filter. This approach is expressed in the following objective function [5]:

(17) $$J_{\text{FCM}\_S'}=\overset{c}{\underset{j=1}{\sum }}\;\overset{n}{\underset{i=1}{\sum }}\mu_{ij}^{b}{\left\|x_{i}-m_{j}\right\|}^{2}+\alpha \overset{c}{\underset{j=1}{\sum }}\;\overset{n}{\underset{i=1}{\sum }}\mu_{ij}^{b}{\left\|x_{i}^{\wedge }-m_{j}\right\|}^{2},$$

where $x_{i}^{\wedge }$ is a mean or median of neighboring pixels lying within a window around *x_i_*. Here, we modify (17) by substituting $x_{i}^{\wedge }$ with denoising molecular image data $x_{i}^{*}$ . The objective function for the FCM with spatial constraints (called FCM_ *S* later) is given by

(18) $$J_{\text{FCM}\_S}=\overset{c}{\underset{j=1}{\sum }}\;\overset{n}{\underset{i=1}{\sum }}\mu_{ij}^{b}{\left\|x_{i}-m_{j}\right\|}^{2}+\alpha \overset{c}{\underset{j=1}{\sum }}\;\overset{n}{\underset{i=1}{\sum }}\mu_{ij}^{b}{\left\|x_{i}^{*}-m_{j}\right\|}^{2}.$$

### 4.3. 2DFCM

Equation (18) introduces spatial constraints into the clustering procedure. However, the classification result of (18) still solely depends on the intensity distribution of the image, which makes it sensitive to intensity variations within a turbid tissue. With the texture information obtained by the Gabor wavelet bank, the two-dimensional fuzzy C-Means (2DFCM) algorithm is constructed by integrating both the intensity and the texture information. Suppose that the texture feature image is *T* = {*t*_1_,*t*_2_, … ,*t_n_*}, the objective function of 2DFCM can be expressed as

(19) $$\begin{array}{ll} J_{2\text{DFCM}} & =\overset{c}{\underset{j=1}{\sum }}\;\overset{n}{\underset{i=1}{\sum }}\mu_{ij}^{b}{\left\|x_{i}-m_{j}\right\|}^{2}+\alpha \overset{c}{\underset{j=1}{\sum }}\;\overset{n}{\underset{i=1}{\sum }}\mu_{ij}^{b}{\left\|x_{i}^{*}-m_{j}\right\|}^{2}\\ & \;+\beta_{i}\overset{c}{\underset{j=1}{\sum }}\;\overset{n}{\underset{i=1}{\sum }}\mu_{ij}^{b}{\left\|t_{i}-v_{j}\right\|}^{2}. \end{array}$$

The influence of the texture characterization imposed on the clustering procedure can be controlled by a constant vector *β_i_* (*i* = 1, … ,*n*) ; the prototype of texture image data is represented by *v_j_* (*j* = 1, … ,*c*). The choice of *β_i_* is based on the following principle. If *t_i_* is large, implying the texture energy is dominant, and *β_i_* should be large; if *t_i_* is small, implying the texture energy is weak, and *β_i_* should be also small. The *β_i_* is determined by *β_i_* = *β_ti_*/max(*T*), where *B* is a constant and its optimized value is determined by “trial-and-error” technique (see Section 5 for details).

The optimization problem under the constraint of *U* as stated in (15) can be solved using one Lagrange multiplier:

(20) $$\begin{array}{ll} F & =\overset{c}{\underset{j=1}{\sum }}\;\overset{n}{\underset{i=1}{\sum }}\mu_{ij}^{b}{\left\|x_{i}-m_{j}\right\|}^{2}+\alpha \overset{c}{\underset{j=1}{\sum }}\;\overset{n}{\underset{i=1}{\sum }}\mu_{ij}^{b}{\left\|x_{i}^{*}-m_{j}\right\|}^{2}\\ & \;+\beta_{i}\overset{c}{\underset{j=1}{\sum }}\;\overset{n}{\underset{i=1}{\sum }}\mu_{ij}^{b}{\left\|t_{i}-v_{j}\right\|}^{2}+\lambda \left(1-\overset{c}{\underset{j=1}{\sum }}\mu_{ij}\right). \end{array}$$

Taking the derivative of *F* with respect to *μ_ij_* and setting the result to zero, we can obtain an equation for *μ_ij_* with unknown

(21) $$\mu_{ij}={\left\{\frac{\lambda }{b[{\left(x_{i}-m_{j}\right)}^{2}+\alpha {\left(x_{i}^{*}-m_{j}\right)}^{2}+\beta_{i}{\left(t_{i}-v_{j}\right)}^{2}]}\right\}}^{1/(b-1)}.$$

Utilizing the constraint of *U* can be solved as

(22) $$\lambda ={\left\{\sum_{k=1}^{c}{\left\{b\left[{\left(x_{i}-m_{j}\right)}^{2}+\alpha {\left(x_{i}^{*}-m_{j}\right)}^{2}+\beta_{i}{\left(t_{i}-v_{j}\right)}^{2}\right]\right\}}^{1/\left(b-1\right)}\right\}}^{b-1}.$$

Substituting (22) into (21), a necessary condition for (19) to be at a local minimum will be obtained:

(23) $$\mu_{ij}=\frac{{\left[{\left(x_{i}-m_{j}\right)}^{2}+\alpha {\left(x_{i}^{*}-m_{j}\right)}^{2}+\beta_{i}{\left(t_{i}-v_{j}\right)}^{2}\right]}^{-1/(b-1)}}{\overset{c}{\underset{k=1}{\sum }}{\left[{\left(x_{i}-m_{k}\right)}^{2}+\alpha {\left(x_{i}^{*}-m_{k}\right)}^{2}+\beta_{i}{\left(t_{i}-v_{k}\right)}^{2}\right]}^{-1/(b-1)}}.$$

Similarly, zeroing the derivative of *F* with respect to *m_j_* and *v_j_*, we have

(24) $$m_{j}=\frac{\overset{n}{\underset{i=1}{\sum }}{\mu_{ij}^{b}}_{}(x_{i}+\alpha x_{i}^{*})}{(1+\alpha )\overset{n}{\underset{i=1}{\sum }}\mu_{ij}^{b}},\;v_{j}=\frac{\overset{n}{\underset{i=1}{\sum }}\mu_{ij}^{b}t_{i}}{\overset{n}{\underset{i=1}{\sum }}\mu_{ij}^{b}}.$$

### 4.4. Implementation of 2DFCM

For the 2DFCM, the number of prototypes (*c*) and the initial centroids (***M*** = {(*m_j_*, *v_j_*) | *j* = 1, … ,*c*}) ought to be known at the beginning of iterative procedures. A maximum likelihood approach by processing and analyzing the two-dimensional (2D) histogram of *X* and *T* is used to estimate *c* and **M**. The number of prototypes (*c*) and initial prototypes (**M**) is estimated by following steps.

1. Count the number of peaks in the 2D histogram and record it as *PeakNum^prev^*.
2. Filter the histogram using a five-by-five Gaussian filter with zero mean and a standard deviation of 0.6.
3. Pick peak points in the 2D histogram and record the number of peaks using *PeakNum^next^*. Calculate *PeakSub=PeakNum^next^−PeakNum^prev^*, and *PeakNum^prev^=PeakNum^next^*.
4. If *PeakSub* < 1, then go to step (5); if *PeakSub* ≥1, then go to step (2);
5. The *c* is estimated as the number of peaks existing in the filtered 2D histogram and the locations of *c* peaks found are used as the initial centroids **M**.

The procedure of 2DFCM can be summarized in the following steps.

1. Filter the image using GNF followed by SRAD to generate the denoising data X*.
2. Filter the image using Gabor wavelet band and compute the texture feature image *T*.
3. Formulate the 2D histogram using the denoising data X* and the texture feature image *T*. Estimate the number of clusters (*c*) and initial prototypes (**M**).
4. Repeat the following steps until the centroids variation is less than 0.001.
  - Update the membership function matrix using (23).
  - Update the centroids using (24).
  - Calculate the centroids variation between before updating and after updating.

## 5. EXPERIMENTAL RESULTS AND DISCUSSIONS

We perform experiments on a PC with 2.0 GHz Pentium processor using Visual C++ 6.0. To illustrate the performance of the 2DFCM, we first test it using simulated molecular images from which the ground truth data is available. Simulated molecular images are obtained by using MOSE (Monte Carlo optical simulation environment) [16–18] developed by Bioluminescence Tomography Lab, Department of Radiology and Department of Biomedical Engineering, University of Iowa (http://radiology.uiowa.edu/). MOSE is based on Monte Carlo method to simulate bioluminescent phenomena in the mouse imaging and to predict bioluminescent signals around the mouse.

The optimized *α* and *B* in the 2DFCM should be obtained by “trial-and-error” technique. We first choose an appropriate value for *α* based on the segmentation performance of the FCM with spatial constraints (FCM_*S*) (the objective function is formularized as (18). We take a group of values for *α* ranging from 0.25 to 6 to test the misclassification rate. With the increasing of *α*, the number of misclassified pixels reduces. However, after *α* exceeds 3, the segmentation performance of the FCM_*S* has no apparent changes. Therefore, we set *α* = 3.5 in our study, which is a value that can produce steady and good results. Then, we choose an appropriate value for *B* based on the segmentation performance of the 2DFCM. We also take a group of values for *B* ranging from 5 to 80 to test the misclassification rate. After *B* exceeds 36, the segmentation performance of the 2DFCM has no apparent changes. Therefore, we set *B* = 36 in our work, which gives steady and satisfactory results. The computation time of the proposed algorithm on an image of 128 × 128 is approximately 12 seconds. About two thirds of total time are consumed in texture characterization based on Gabor wavelet.

**Figure 3:**
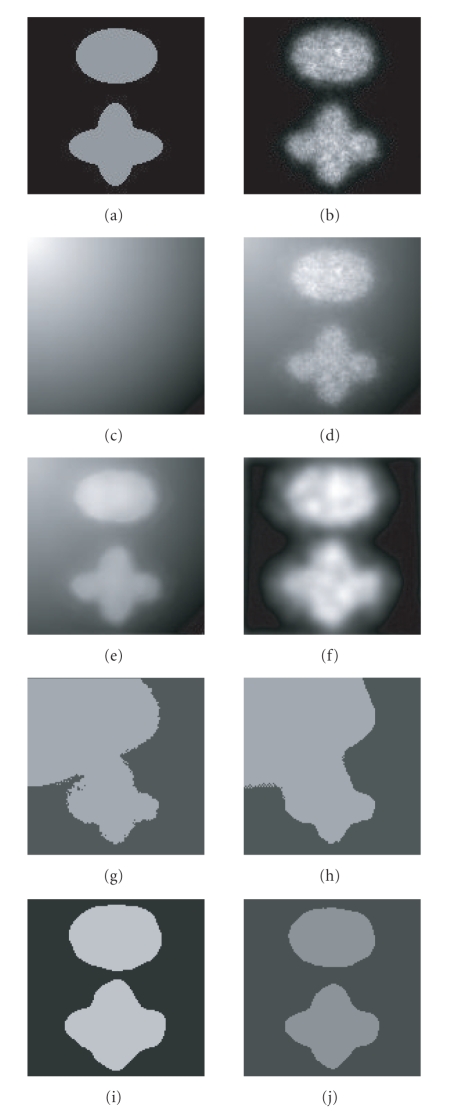
Segmentation results on the first synthetic image: (a) the ground truth image; (b) the synthetic image generated by MOSE; (c) the intensity inhomogeneity model; (d) the synthetic image corrupted by the intensity inhomogenetiy; (e) the denoising result with the GNF plus SRAD; (f) the texture feature image; (g) the FCM result on (d); (h) the FCM_*S* result on (d); (i) the FCM result on texture feature image; (j) the 2DFCM result.

The first example is applying algorithms to a synthetic cellular image and comparing the 2DFCM with other three algorithms, including the FCM on the original image, the FCM with spatial constraints, and the FCM on the texture feature image. The model to generate synthetic molecular images is illustrated in Figure 3(a). The simulated molecular image (128 × 128) corresponding to Figure 3(a) is shown in Figure 3(b). Then Figure 3(b) is corrupted by the intensity inhomogeneity (as shown in Figure 3(c)) to generate the final synthetic image (as shown in Figure 3(d)). Figure 3(e) shows the image filtered by the GNF plus SRAD. Figure 3(f) shows the texture feature image obtained by the Gabor wavelet bank. Figures 3(g)–3(j) give the segmentation results of the FCM on the original image (Figure 3(d)), the FCM with spatial constraints, the FCM on the texture feature image (Figure 3(f)), and the 2DFCM, respectively. We quantify the algorithm performance in terms of three parameters defined as follows:

(25) $$\begin{array}{ll} \text{SA} & =\frac{N_{\text{CORRECT}}}{N_{\text{TOTAL}}},\\ \text{US} & =\frac{N_{fp}}{N_{n}},\\ \text{OS} & =\frac{N_{fn}}{N_{p}}. \end{array}$$

SA represents the total segmentation accuracy; US is the under segmentation rate; OS denotes the over segmentation rate. *N*_CORRECT_ is the number of correctly classified pixels; *N*_TOTAL_ is the total number of pixels; *N_fp_* is the number of pixels that do not belong to the class and are segmented into this class; *N_fn_* is the number of pixels that belong to the class and are not segmented into the class; *N_p_* is the number of all pixels that belong to the class; *N_n_* is the number of all pixels that do not belong to the class. There are totally four algorithms that are compared in our experiments. Table 1 gives the SA, US, and OS comparisons among the four algorithms, correspondingly.

**Figure 4:**
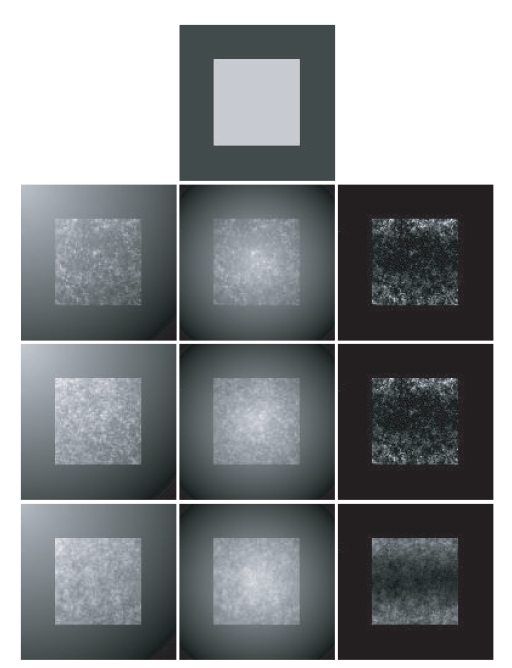
Synthetic images under different imaging conditions. First row: the ground truth image. From the second row to bottom: synthetic images with different photons density along the vertical direction and different types of intensity inhomogeneity along the horizontal direction.

To further test the segmentation performance of the proposed method, a group of synthetic images under different imaging conditions are utilized. Nine synthetic images are shown in Figure 4. These images are organized into the form with different photons density along the vertical direction and different types of intensity inhomogeneity along the horizontal direction. Table 2 summarizes the segmentation accuracy of the FCM on the original image, the FCM with spatial constraints, the FCM on the texture feature image, and the 2DFCM, respectively.

**Figure 5:**
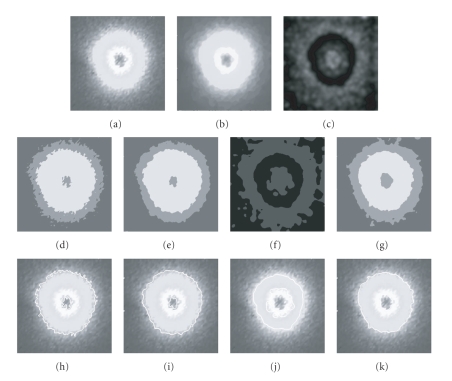
Segmentation results on a real molecular image: (a) the original image; (b) the denoising result with the GNF plus SRAD; (c) the texture feature image; (d) the FCM result on (a); (e) the FCM_*S* result on (a); (f) the FCM result on the texture feature image; (g) the 2DFCM results; (h)–(k) the contours obtained from (d) to (g) superimposed on the original image, respectively.

The second example is applying the algorithms to a real-molecular image(256 × 256) (as shown in Figure 5(a)). Figures 5(b) and 5(c) show the denoising result of the GNF plus SRAD, and the texture feature image obtained by the Gabor wavelet bank, respectively. Figures 5(d)–5(g) illustrate the segmentation results of the FCM on the original image, the FCM with spatial constraints, the FCM on the texture feature image, and the 2DFCM, respectively. In order to illustrate the segmentation results clearly, the contours of the interest of region in the classification image are extracted and superimposed on the original image. Figures 5(h)–5(k) give the contour comparisons. It can be seen from Figure 5(a) that the middle of the tissue appears homogeneously bright. However, the molecular concentration decreases in the boundary area, which leads to the intensity variation near the boundary. The conventional FCM on the original image and the FCM with spatial constraints produce undersegmentation results and the FCM on the texture feature image shows oversegmentation.

From the experimental results, we can see that the denoising effects of the GNF plus SRAD are satisfactory. The Gabor wavelet bank can represent the texture information in the molecular image without being disturbed by the intensity variation. The FCM produces the worst result due to the fact that no spatial constraints are used in it. The FCM with spatial constraints produces more smoothed segmentation results than the FCM. However the intensity inhomogeneity makes the segmentation result degenerate. Since the 2DFCM utilizes both the intensity and texture information simultaneously, it produces more satisfactory results than other methods.

## 6. CONCLUSIONS

In this paper, we have developed a novel algorithm based on the fuzzy clustering for the molecular image segmentation. Considering that there are two disadvantages for the conventional FCM in the image segmentation, its successful employment in the molecular image segmentation requires overcoming nonrobust factors by introducing spatial constraints and the texture feature of images into the clustering. To alleviate noises in molecular images, a denoising method combining GNF plus SRAD is proposed. We use the denoising data obtained by GNF plus SRAD to compose spatial constraints for the new 2DFCM. By utilizing the Gabor wavelet representation and the texture energy characterization, the texture feature that is insensitive to intensity variations is introduced into the 2DFCM. Quantitative evaluation demonstrates the superiority of the 2DFCM over the conventional FCM in the molecular image segmentation.

## Figures and Tables

**Table 1:** SA, US, and OS of the three-conventional FCM and 2DFCM on Figure 3.

Parameter	The FCM on original image	The FCM with spatial constraints	The FCM on texture image	The 2DFCM
SA	0.72	0.71	0.91	0.97
US	1.12	1.16	0.35	0.13
OS	0.01	0.01	0	0

SA: segmentation accuracy.

US: undersegmentation rate.

OS: oversegmentation rate.

**Table 2:** SA of the three-conventional FCM and 2DFCM on images with different imaging conditions.

Parameter	The FCM on original image	The FCM with spatial constraints	The FCM on texture image	The 2DFCM
SA	0.87 ± 0.15	0.86 ± 0.14	0.93 ± 0.03	0.96 ± 0.03
